# Genome-wide characterization of the biggest grass, bamboo, based on 10,608 putative full-length cDNA sequences

**DOI:** 10.1186/1471-2229-10-116

**Published:** 2010-06-18

**Authors:** Zhenhua Peng, Tingting Lu, Lubin Li, Xiaohui Liu, Zhimin Gao, Tao Hu, Xuewen Yang, Qi Feng, Jianping Guan, Qijun Weng, Danlin Fan, Chuanrang Zhu, Ying Lu, Bin Han, Zehui Jiang

**Affiliations:** 1 Chinese Academy of Forestry, Wanshou Shan, Beijing 100091, PR China; 2 National Center for Gene Research & Shanghai Institute of Plant Physiology and Ecology, Shanghai Institutes for Biological Sciences, Chinese Academy of Sciences, Shanghai 200233, PR China; 3 International Network for Bamboo and Rattan, 8 Fu Tong Dong Da Jie, Chaoyang District, Beijing 100102, PR China; 4 Beijing Institute of Genomics, Chinese Academy of Sciences, Beijing 100029, PR China

## Abstract

**Background:**

With the availability of rice and sorghum genome sequences and ongoing efforts to sequence genomes of other cereal and energy crops, the grass family (Poaceae) has become a model system for comparative genomics and for better understanding gene and genome evolution that underlies phenotypic and ecological divergence of plants. While the genomic resources have accumulated rapidly for almost all major lineages of grasses, bamboo remains the only large subfamily of Poaceae with little genomic information available in databases, which seriously hampers our ability to take a full advantage of the wealth of grass genomic data for effective comparative studies.

**Results:**

Here we report the cloning and sequencing of 10,608 putative full length cDNAs (FL-cDNAs) primarily from Moso bamboo, *Phyllostachys heterocycla *cv. *pubescens*, a large woody bamboo with the highest ecological and economic values of all bamboos. This represents the third largest FL-cDNA collection to date of all plant species, and provides the first insight into the gene and genome structures of bamboos. We developed a Moso bamboo genomic resource database that so far contained the sequences of 10,608 putative FL-cDNAs and nearly 38,000 expressed sequence tags (ESTs) generated in this study.

**Conclusion:**

Analysis of FL-cDNA sequences show that bamboo diverged from its close relatives such as rice, wheat, and barley through an adaptive radiation. A comparative analysis of the lignin biosynthesis pathway between bamboo and rice suggested that genes encoding caffeoyl-CoA O-methyltransferase may serve as targets for genetic manipulation of lignin content to reduce pollutants generated from bamboo pulping.

## Background

We rely on grasses more than any other groups of plants for food and potential renewable energy. With the number of genome sequences growing much more rapidly than any other plant family, the grass family (Poaceae) becomes an ideal system for comparative studies of gene and genome structure and function [1-6]. To develop the system, it is critical to accumulate genomic resources for all major lineages of the grass family. The majority of large subfamilies of grasses have already had a great deal of genomic or expressional data available primarily because they possess crop species. The only exception is the subfamily of bamboos, Bambusoideae, which contains more than 1,000 species but has little data available in DNA or protein sequence databases [4,7]. By January of 2009, the number of ESTs deposited in the GenBank ranged from 436,535 to 2,018,337 for rice, wheat, maize, barley, sorghum, sugarcane, and switchgrass, but only 3,087 for bamboo. This creates a major missing link in the grass family for comparative genomics.

More serious than the missing link for comparative analyses is the lack of genomic resources of bamboo. This hampers biological investigations of this group of morphologically and physiologically unique and ecologically and economically important grasses. Unlike the majority of ~10,000 grass species that are herbaceous and occupy open habitats such as grassland, bamboo represents the only major lineage of grasses that lives exclusively in forests and grows large woody culms up to 30 cm in diameter and 12 m in height [8]. In addition to remarkable sizes and woodiness, bamboo has rather striking life history characterized by a prolonged vegetative phase lasting up to more than 100 years before flowering.

With these unique features, bamboos are important components of tropical and subtropical forest ecosystems, especially in Asia, where they have had a long history of being utilized as garden ornamentals and forest products for making construction material, paper pulp, and furniture. With the realization that bamboo produces high-quality fibers and can be harvested repeatedly without severe destruction of the ecosystems, it becomes an increasingly valuable forest product that could replace a substantial portion of tree-based timber and paper pulp plantation. This highlights another important economic value of grasses in addition to food and renewable energy.

In this study, we cloned and sequenced more than ten thousand putatively unique FL-cDNA derived primarily from vegetative tissues of Moso bamboo, *Phyllostachys heterocycla *cv. *Pubescens*, a large woody bamboo with the highest ecological, economic, and cultural values of all bamboos in Asia and accounting for ~70% of total area of bamboo growth and 5 billion US dollars of annual forest production in China [9] (Additional file 1). The sequences reported in this study represent the third largest collection of FL-cDNA sequences of all plant species, only smaller in number than those of Arabidopsis and rice. It provides the first large sequence dataset for studying the structure and function of a substantial portion of bamboo genes, and fills the gap in the grass family for comparative genomics. A multiple-gene phylogeny inferred from these data shed light on the evolutionary relationships within the grass family. Comparative analyses of the bamboo sequence data with those of rice, barley, and wheat yielded new insights into gene evolution associated with rapid and marked phenotypic and ecological divergence between bamboo and closely related grasses. Comparison of bamboo cDNAs and rice genes involved in lignin biosynthesis suggested that genes encoding caffeoyl-CoA O-methyltransferase may serve as effective targets for genetic manipulation of lignin content to reduce pollutants generated from bamboo pulping.

## Results

### Structure of bamboo cDNA sequences

From screening and sequencing five cDNA libraries, a total of 37,797 5'-end sequences comprised of at least 100 consecutive nucleotides with a Phred score above 20 were obtained and assembled into 10,669 singletons and 3,373 contigs. The average and maximal sizes of contigs were 8 and 288 cDNA clones, respectively. All singletons and one representative of each contig were fully sequenced and further filtered for redundancy, which yielded 10,608 putatively unique FL-cDNA sequences. The average length of these FL-cDNAs was 1,092 bp, and 8,695 (82%) cDNAs had open reading frames (ORFs) longer than 100 amino acids, similar to 87% in soybean [10] and 91% in poplar [11].

The characteristics of these 8,695 cDNAs were compared with 37,165 FL-cDNAs of rice and 17,390 FL-cDNAs of Arabidopsis also with predicted ORFs longer than 100 amino acids. The mean GC content of the 8,695 bamboo FL-cDNAs is 54.0%, ranging from 30.1 to 72.1%, with the highest GC content of 69.2% at the third codon position (Table 1). The mean GC content also varies among 5' untranslated regions (UTRs), ORFs, and 3' UTRs, with that in 3' UTRs lower than the other two regions (Additional file 2). Bamboo codon usages are calculated and compared with those of rice and Arabidopsis (Additional file 3).

**Table 1:** Mean percentage of GC content of bamboo, rice, and Arabidopsis FL-cDNAs.

	Number	Length	5'UTR	3'UTR	ORF	1st	2nd	3rd
Bamboo	8,695	54.0	56.8	42.3	58.1	59.1	46.0	69.2
Rice	37,165	52.0	54.0	41.4	55.2	57.9	45.1	62.7
Arabidopsis	17,390	43.5	42.9	38.6	45.6	51.4	41.8	43.5

Codon usage is compared in 5' and 3' UTRs, and the three codon positions of ORFs.

**Figure 1:**
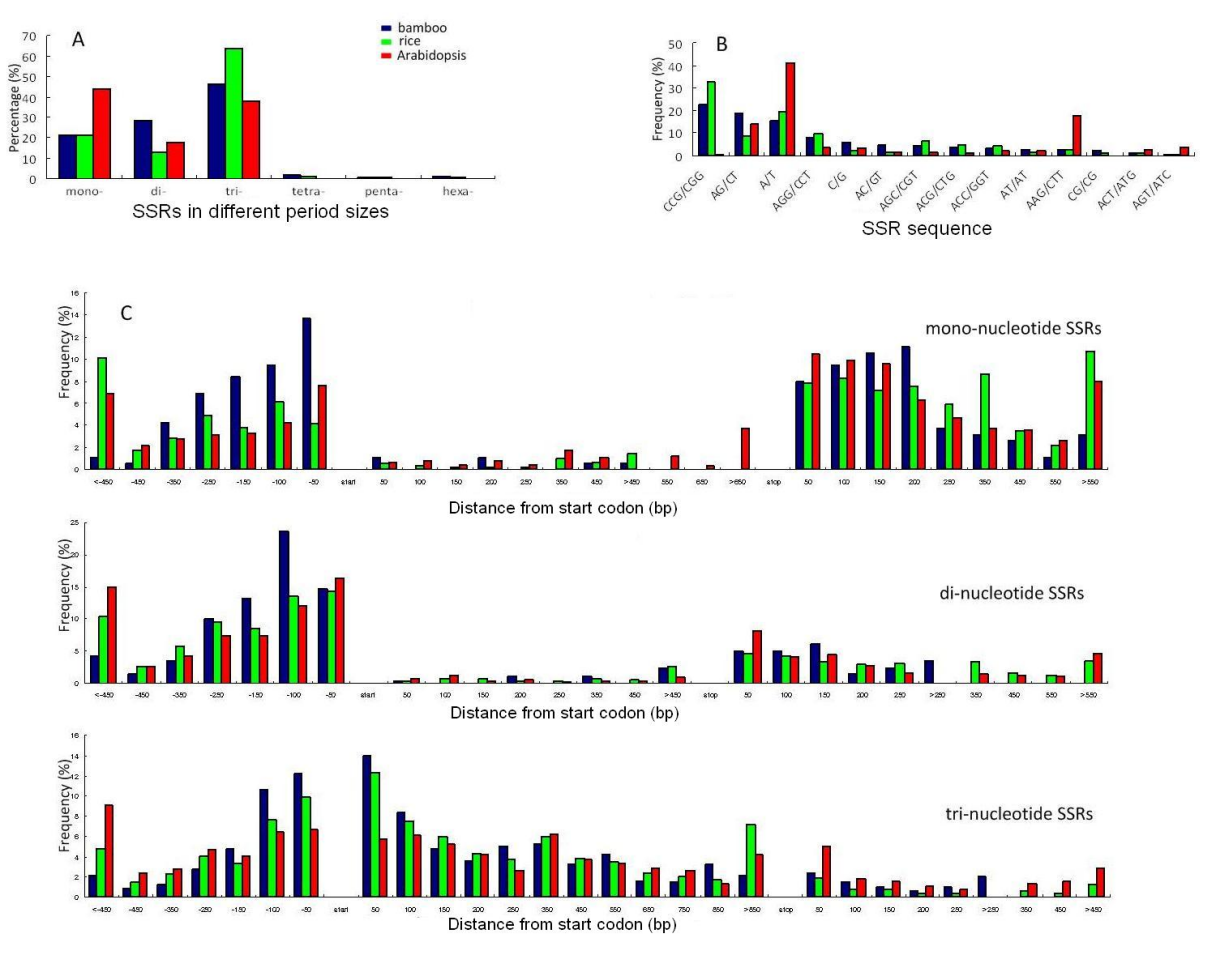
**Types and frequency of occurrence of simple sequence repeats (SSRs) in cDNAs of bamboo, rice, and Arabidopsis**. (A) Percentage of SSRs in different period sizes. Relative abundance of different types of SSRs is classified according to repeat length. (B) Frequency of SSR sequences in different types. Relative frequency of 14 most abundant repeat sequences of SSRs is shown. (C) Frequency and position of mono-, di-, and tri-nucleotide SSRs. Frequency distribution of mono-, di-, and tri-nucleotide SSRs are calculated along the predicted transcribed regions.

Approximately 24% of the 8,695 bamboo cDNAs contain at least one single sequence repeat (SSR), which is the same as the percentage found in Arabidopsis and lower than the 44% found in rice. The frequencies of specific types of SSRs found in the bamboo, rice, and Arabidopsis cDNA sequences are illustrated in Figure 1, of which mono-, di-, and tri-nucleotide repeats are predominant. With regard to the location of SSRs, mono-nucleotide repeats are most frequent in 5' and 3' UTRs, di-nucleotide repeats are most frequent in 5' UTRs, and tri-nucleotide repeats have a relatively high frequency in ORFs (Figure 1).

### Alternative splicing, natural antisense transcripts, and transposable elements

With the absence of bamboo genome sequences, we identified putative alternative splicing (AS) patterns based on six major AS types and the GT-AG rule of exon/intron boundaries (Additional file 4A). A total of 304 cDNA groups were predicted to have AS, of which 138 were putative intron retention (IntronR) or Alternative position (AltP) types, including 6 with GC-AG exon/intron boundaries, and 92, 29, 15 and 14 were putative ExonO, ExonS, AltA and AltD types, respectively (Additional file 4B). Of these, 16 cDNA groups have more than one AS patterns.

Natural antisense transcripts (NATs) overlap partially the sequences of other endogenous sense transcripts in the opposite direction. Overlapping genes involving exon regions are known as cis-encoded natural sense-antisense gene pairs [12,13]. Of the 10,608 FL-cDNAs, 25 were identified to be sense-antisense transcripts (Additional file 5). The average overlapping length was 467 bp, with a range from 73 to 960 bp. Thirteen had predicted ORFs longer than100 amino acid and 10 had predicted ORFs between 30 and 100 amino acid, while one failed to have predicted ORFs.

BLASTX searches of the bamboo cDNAs against plant transposable element (TE) database revealed that 53 of 10,608 cDNA or 0.5% showed significant match to TEs, of which 32, 10, and 10 were homologous to polyproteins, transposases of class-I DNA elements (Mutators), and class-II RNA elements (non-long terminal repeat, non-LTR), respectively. One matched to a TE in the reverse orientation, which might be a result of genomic contamination or read-through from neighboring retrotransposons [14].

### Functional annotation and genes involved in lignin biosynthesis

A total of 9,496 (89.5% of ) bamboo FL-cDNAs matched proteins of known or unknown functions based on BLASTX against NCBI non-redundant database (nrDB). A total of 5,404 (50.9%) cDNAs showed similarity with PFAM protein families in the InterPro database, of which those belonging to the top 20 most abundant families are shown in Additional file 6. A total of 387 (3.6%) cDNAs were predicted to be putative transcription factors according to nrDB, of which those in the top 10 most abundant classes are shown (Additional file 7). The InterPro Gene Ontology (GO) assignment identified 4,594 cDNA with GO terms associated with molecular functions in 11 categories (Additional file 8).

To take a snapshot on the functional representation of the cDNAs, we identified from the 10,608 FL-cDNAs genes encoding nine key enzymes involved in lignin biosynthesis (KEGG PATH: ko00940, http://www.genome.jp/kegg/. A total of 35 cDNAs were identified, with at least one and as many as nine cDNAs found for each of the nine enzymes (Table 2). For each enzyme, we compared the number of putatively unique bamboo cDNAs isolated in this study with the number of rice genes identified from the genome sequences. The ratio of the number of rice genes to that of bamboo cDNAs showed a wide range of variation from 1.1 to 13, with an average of 5 (Table 2). For each enzyme, a phylogeny was inferred based on the aligned nucleotide sequences between the bamboo cDNAs and rice genes (Additional file 9).

**Table 2:** Number of bamboo FL-cDNAs and number of genes found in the rice genome that encode nine key enzymes in the lignin biosynthesis pathway.

Enzymes	Bamboo cDNAs	Rice genes	Ratio (R/B)
4-coumarate-CoA ligase (4CL)	2	26	13.0
Caffeoyl caffeoyl-CoA O-methyltransferase (CCoAOMT)	9	10	1.1
Cinnamoyl-CoA reductase (CCR)	7	18	2.7
Caffeic acid O-methyltransferase (COMT)	2	10	5.0
Cinnamate-4-hydroxylase (C4H)	1	4	4.0
Cinnamoyl alcohol dehydrogenase (CAD)	6	21	3.5
Laccase	5	23	4.6
5-hydroxyconiferyl aldehyde O-methyltransferase (AldOMT)	1	7	7.0
3-deoxy-D-arabino-heptulosonate 7-phosphate synthase (DAHPS)	2	8	4.0

The ratio of the number of rice genes to the number of bamboo cDNAs (R/B) is calculated.

### Comparative and phylogenetic analyses with other grasses

Sequence similarity search was conducted between bamboo and eight other grasses with the largest amount data available in TIGR Plant Transcript Assemblies. They represent three of the four large subfamilies with more than 100 species: rice (*Oryza sativa*) from the subfamily Ehrhartoideae with >100 species; wheat (*Triticum aestivum*), barley (*Hordeum vulgare*), and *Brachypodium distachyon *from Pooideae with >3,000 species; and maize (*Zea mays*), sorghum (*Sorghum bicolor*), sugarcane (*Saccharum officinarum*), and switchgrass (*Panicum virgatum*) from Panicoideae with >3,000 species. As bamboo represents the subfamily Bambusoideae with >1,000 species, Chloridoideae with >1,000 species is the only large subfamily not represented in this study due to the lack of sufficient amount of genomic data for informative comparative analysis (maximal ~20,000 ESTs for a single species).

Search for nucleotide sequence similarity with a relatively high stringency (E-value < 1e-10 in BLASTn) showed that 49.2% of bamboo cDNAs had similarity hits to rice transcripts, the highest among the grasses (Additional file 10). This compares to 81.2% and 58.1% of cDNAs that had similarity hits to rice and Arabidopsis, respectively, when the search was conducted for amino acid sequences under a less stringent condition (E-value < 1e-6 in tBLASTx) [15]. The higher proportion of bamboo cDNAs had hits to Arabidopsis than *Branchypodium *and switchgrass was due to the relatively small number of transcripts available for these two grasses. Similarity search was also conducted between the bamboo FL-cDNAs and the genome sequences of rice, sorghum, Arabidopsis, and poplar with both nucleotide and amino acid sequences under different stringencies. The proportions of similarity hits were somewhat lower than those hit the transcript database (Additional file 11).

**Figure 2:**
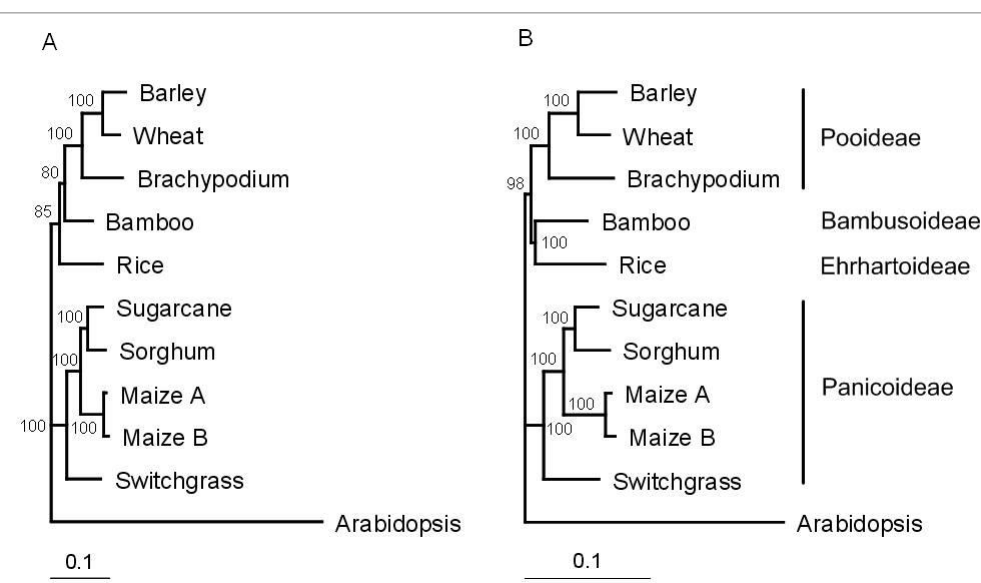
**Phylogeny of grasses inferred from concatenated alignment of 43 putative orthologous cDNA sequences**. (A) Tree inferred from maximal likelihood method. Bayes inference yielded the same topology. (B) Tree inferred from neighbor joining method. Branch length is proportional to estimated sequence divergence measured by scale bars. Numbers associated with branches are bootstrap percentages. Arabidopsis was used as outgroup. Subfamily affiliation of the grasses is indicated at right.

To find a set of orthologous sequences for phylogenetic analysis of the grass family, we further increased the search stringency by keeping those with one-to-one match in nucleotide sequences between bamboo and another species (see Methods). With this conservative criterion, we identified 43 putative orthologs among the nine grass species plus Arabidopsis serving as an outgroup (Additional file 12). The concatenated alignment of these 43 sequences was 38,418 bp long, and was subjected to phylogenetic analyses using three methods. The resulting phylogenetic trees differed in the relationship between subfamilies, Bambusoideae (bamboo), Pooideae (barley, wheat, *Brachypodium*), and Ehrhartoideae (rice) (Figure 2). The trees generated by maximum likelihood and Bayes inference were the same and supported a closer relation between Bambusoideae and Pooideae, which then formed a sister group with Ehrhartoideae. The tree resulted from the Neighour Joining analysis, however, supported a closer relationship between Bambusoideae and Ehrhartoideae. Each of the three trees was fully resolved and well supported, with all branches including those incongruent between trees supported by bootstrap values higher than 80% or Bayes probability of 1.0.

**Figure 3:**
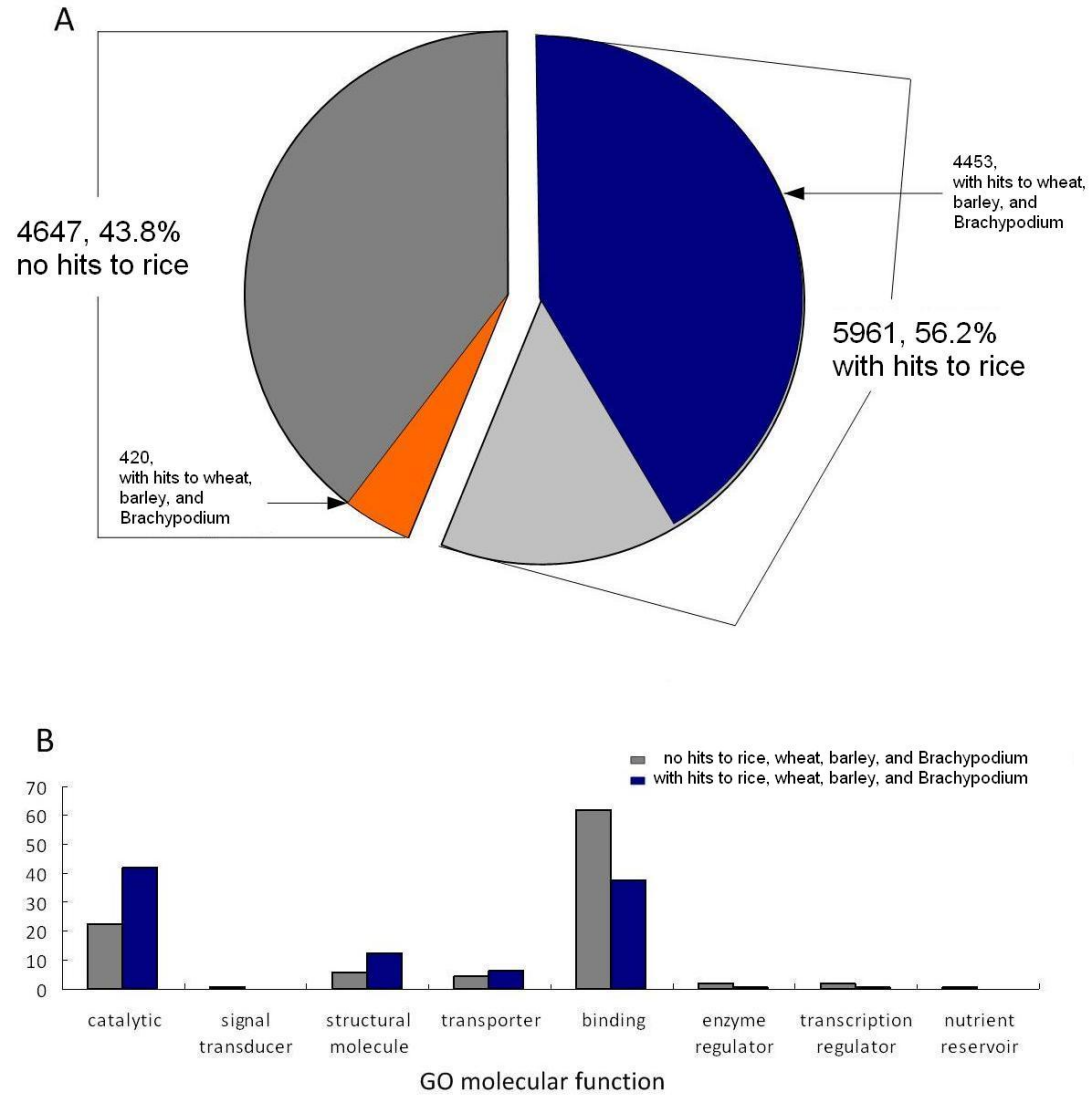
**Bamboo cDNA similarity comparison and functional classification**. (A) Similarity search of bamboo sequence against closely related grasses. Right half of the pie, the proportion of bamboo cDNAs with homologs in rice, of which the proportion that also hit barley, wheat, and *Brachypodium *is colored blue. Left half, bamboo cDNAs with no homologs found in rice, of which the proportion that also had no hit in the barley, wheat, and *Brachypodium *is colored gray. (B) Functional classification of bamboo cDNAs with and without homologs with other grasses. Blue bars represent cDNAs from the blue portion of the pie shared by all grasses compared, and gray bars represent cDNAs from the gray portion of the pie which is presumably bamboo unique.

The close relationship of Bambusoideae with Pooideae and Ehrhartoideae provides an opportunity for comparing gene evolution between these three groups of grasses. Given the wealthy of rice genome data, we examined at first the proportion of bamboo FL-cDNAs that matched rice databases at the nucleotide sequence level. A total of 5961 or 56.2% of bamboo cDNAs matched rice sequences, while 43.2% had no hit to rice sequences (Figure 3A). We then conducted the similar search against databases of wheat, barley, and *Brachypodium *and found that 4453 or 42% of bamboo cDNAs that had homologs with rice also had homologs in wheat, barley, and *Brachypodium*. Of 4,647 bamboo cDNAs that did not match any of the rice sequences, 420 had homologs with wheat, barley, and *Brachypodium*, which left 4227 or 39.8% of bamboo cDNAs without homologs found in any of the closely related species.

We then compared the functional classification between two groups of bamboo cDNAs, one including shared homologs with other two subfamilies and the other being unique to bamboo (Figure 3B). It is noteworthy that shared cDNAs are relatively abundant in catalytic molecules, structural molecules, and transporters. In contrast, cDNAs unique to bamboo are relatively abundant in groups with binding activities and regulatory functions.

**Figure 4:**
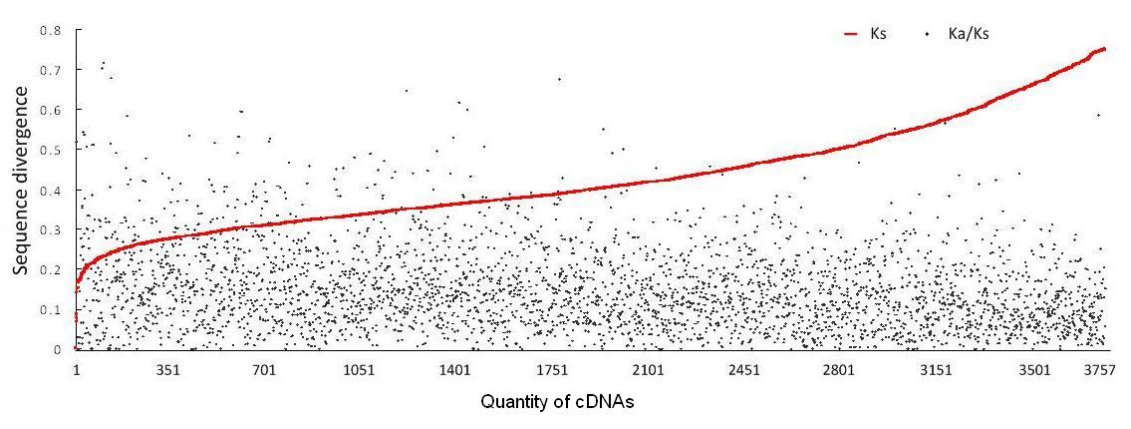
**Distribution and functional classification of bamboo cDNAs according to sequence divergence compared with rice cDNAs**. Red curve shows distribution of bamboo cDNAs according to synonymous divergence (*K*_*S*_) with rice homologs. Dots, corresponding nonsynonymous divergence (*K*_*A*_). Only cDNAs with *K*_*S *_< 0.75 are shown.

We then compared the bamboo cDNAs with the large collection of rice FL-cDNAs. For 5,961 pairs of aligned sequences between bamboo and rice, synonymous (*K*_*S*_) and nonsynonymous (*K*_*A*_) sequence divergences were calculated (Additional file 13). For previously identified 43 sequences that were presumably orthologous among grasses, the average *K*_*S *_between bamboo and rice was 0.368. We thus examined sequence pairs with *K*_*S *_< 0.75, which should encompass the vast majority of sequences pairs that are likely orthologous between bamboo and rice (Figure 4). This included 3,757 bamboo-rice pairs, of which only four had *K*_*A*_/*K*_*S *_>1.0. The chromosomal distribution of rice homologs with different divergence of bamboo homologs suggested that the isolated bamboo genes in this study were likely random arcross the genome (figures shown in additional file 14).

### Databasing FL-cDNAs and ESTs of Moso bamboo and EST redundancy analysis

To facilitate the access and utilization of the bamboo cDNA sequences, we developed a Moso bamboo cDNA database (MBCD), including 10,608 putative FL-cDNAs and nearly 38,000 ESTs. The database is hosted on a REDHAT LINUX server accessible at the URL http://www.ncgr.ac.cn/MBCD/ (Additional file 15). The source code is available at http://www.ncgr.ac.cn/mbcd/code/ or http://www.ncgr.ac.cn/mbcd/res/down.php The database has the following features: 1) FL-cDNA clone list search. Given cDNA clone ID or NCBI accession number, MBCD will display the cDNA sequences with predicted ORF information, library (tissue) sources, clone ID, Genbank accession numbers, and corresponding ESTs; 2) Sequence search. Blast is provided for sequence alignment search against the cDNA database. Conserved domain search is also available when putative function information (Pfam) is entered; 3) FTP download links. The cDNA sequences, annotations, and corresponding ESTs can be downloaded as compressed files of gzip or bzip; and 4) Other useful web links. Other database websites and research institutes related to bamboo research are conveniently accessible through this website.

Taking advantage of the database, we took a snapshot on the genome redundancy of Moso bamboo by identifying and analyzing EST clusters. BLASTN searches of all ESTs of Moso bamboo were conducted against the FL-cDNAs. We used stringent criteria to identify EST clusters that contained at least 6 homologous ESTs with each sequence polymorphism supported by at least two ESTs. A total of 2,728 clusters were identified, which included 21,497 ESTs.

**Figure 5:**
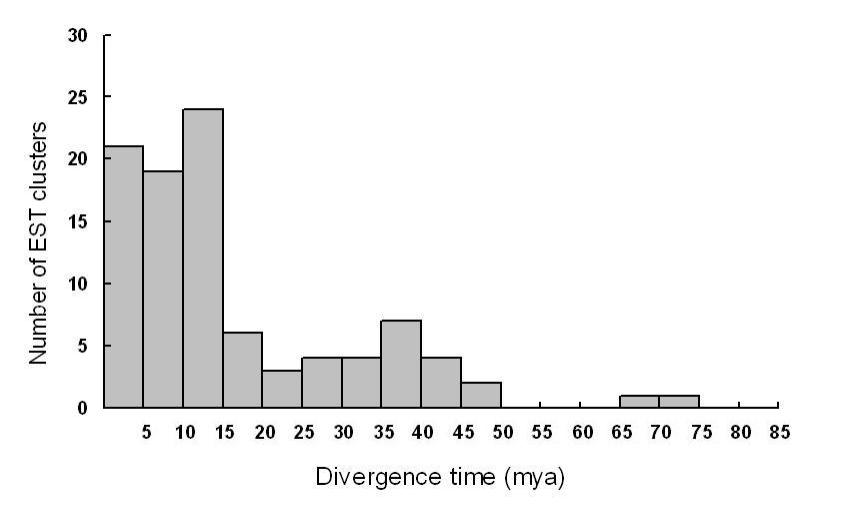
**Distribution of divergence time**. X axis is the divergence time (mya) of homologous pairs, Y axis is the number of detected EST clusters.

To better understand the nature of the redundancy, we calculated sequence divergence and estimated divergence time among members of a random sample of the clusters. To ensure a consistent estimation, only those high-quality ESTs covering the entire coding regions were sampled. This random sample included 215 clusters, of which 100 contained only two types of homologs. We then calculated the synonymous sequence divergence between the two homolog types for the 100 clusters, and estimated the time of divergence using the divergence rate of 6.5 × 10^-9 ^mutations per synonymous site per year [16]. The number of EST clusters with homologs diverged in the range of estimated time intervals is illustrated in Figure 5.

## Discussion

### Bamboo genes and genome

In comparison with a large number of plant ESTs deposited in public database (NCBI ESTs of plants >100 millions), only a small fraction of high-quality sequence-finished FL-cDNAs have been generated. Prior to this study, rice and Arabidopsis were the only plant species with more than 10,000 fully sequenced FL-cDNAs available in the public databases [17-19]. Thus, here we report the third largest FL-cDNA collection of a plant species. Given the estimate that the fully-sequenced genomes of rice and sorghum had approximately 35,000 - 38,000 genes [20,21], the 10,608 putative FL-cDNAs are likely to represent more than a quarter of bamboo genes. One should note this cDNA collection was from various vegetative tissues while the floral tissues were not available for this study because Moso bamboo flowers every 67 years on average. Nevertheless, these data provide a valuable resource for the first glance at bamboo gene diversity and for comparative genomic studies among grasses.

GC content is an important characteristic of genomic composition, reflecting various genome features, such as gene density and structure, codon usage, repeat element distribution, and methylation patterns [15,22,23]. Here we found that bamboo had the highest overall GC content as well as the highest GC content at the third codon position in comparison to rice and Arabidopsis (Table 1; [24,25]). Further, the third codon position of bamboo and rice both displays the highest GC content, followed by the first and then the second positions, whereas Arabidopsis has the highest GC content at the first codon position. With regard to the distribution of GC content in the 5' UTR, ORF, and 3' UTR, we found similarities between the two grasses that differed from Arabidopsis (Additional file 2). The distributions of GC content in the 5' UTR and ORF are rather flat or slightly bimodal for bamboo and rice, but clearly unimodal for Arabidopsis.

The codon usages of bamboo and rice also share considerable similarities in comparison to that of Arabidopsis (Additional file 2). Given the much higher GC content of bamboo and rice cDNAs, it is not surprising that they use G and C more frequently whereas A and T appear at a higher frequency for the same codons in Arabidopsis. There has been evidence that high GC content and corresponding codon usage bias of rice might have influenced the level of gene expression and the mechanisms of gene evolution [26]. The even higher GC content found in Bamboo could have played a role in the genetic and genomic divergence of the large, woody grass.

The type and distribution of SSRs of bamboo are also more similar to those of rice than Arabidopsis. The tri-nucleotide SSRs are more abundant than mono- and di-nucleotide SSRs in bamboo and rice, whereas mono-nucleotide SSRs are the most abundant in Arabidopsis (Figure 1). Of tri-nucleotide SSRs of bamboo and rice that occur predominantly in ORFs, the CCG/CGG type is much more abundant than others. This is perhaps correlated with the higher GC content of the grasses, and may have allowed frequently insertion/deletion of certain amino acids without causing frame shift mutations [27]. Interestingly, bamboo has higher frequency of all three major SSRs in 5' UTRs from the start codon to 150 bp upstream than rice and Arabidopsis, implying that SSRs may have played an important role in regulating gene expression in bamboo.

Alternative splicing may be involved in posttranscriptional regulations that increase protein diversity and affect mRNA stability [28,29]. According to the previous classification of AS [30], we found two types of AS, IntronR and single exon overlapping (sExonO), were more abundant than others. We calculated the length between two continuous exons of each cDNA pair in the IntronR type, which gave an estimate of mean intron size of 218 bp, comparing to 461 bp and 158 bp of the mean intron sizes of rice and Arabidopsis, respectively [19,31].

After the BLASTX searching against NCBI nrDB, 672 bamboo FL-cDNAs had no hit in the database, some of which may be new genes. Functional annotation of transcription factors indicated that ERF was the most abundant among bamboo cDNAs, which was followed by MYB, Zinc finger, WRKY, homeobox, bZIP, bHLH, and NAC. This differs from what was found in a rice cDNA collection where Zinc finger was much more abundant than other transcription factors, whereas ERF was ranked as the third abundant, fewer than Myb [18]. The difference could be due to differential amplification of various families of transcription factors during the evolutionary divergence between bamboo and rice. But it is possible that at least a part of the difference is due to an artifact of cDNA isolation from different tissues at different developmental stages between the two studies.

Because we filtered gene redundancy by selecting one cDNA clone from a contig for full-length sequencing, the sequences of FL cDNAs could not be used to evaluate the genome redundancy. Because the Moso bamboo was considered to be a tetraploid [32], we went back to analyze the ESTs to take a snapshot at the genome redundancy. From a total of 100 sampled EST clusters, the divergence times estimated between two homologous EST types within clusters had an interesting distribution (Figure 5). The abrupt increase in redundancy around 15 million years ago (mya) or more recent is probably a result of genome duplication through polyploid formation. With the EST data, we were unable to determine whether this was an autopolyploidy or allopolyploidy event. In any case, it seems like that the two diploid genomes have diverged for less than 15 million years according to this estimation. The actual divergence time, however, could be longer given that the Moso Bamboo has a longer generation time than most of grasses from which the synonymous substitution rate was calculated.

### Genes involved in lignin biosynthesis

The lignin content of bamboo is comparable to that of woody plants and higher than most herbaceous plants, which has contributed to high culm rigidity that allows bamboo to grow into a large forest [33]. The lignin content of mature culms of Moso bamboo, estimated at about 23% of dry biomass [34], is nearly twice as high as that of rice straw (12.5%, [35]). The high lignin content, however, has a negative impact on paper production from bamboos because removal of lignin during pulping results in hazardous waste. A better under understanding of lignin biosynthesis is important for manipulation lignin content or composition through genetic engineering.

On average, bamboo cDNAs for lignin biosynthesis enzymes isolated in this study are one fifth of the total number of rice genes encoding lignin biosynthesis enzymes. This is not surprising because the cDNAs may represent only one third to one fourth of bamboo genes. From these ratios as well as the phylogenetic inference, it seems likely that there has not been a large-scale duplication of lignin biosynthesis genes in bamboo after it diverged from rice (Additional file 9). Whereas gene duplication might have played a role in altering certain physiological properties, such as drought tolerance in sorghum [21], it was probably not a major reason for the increased lignin content of bamboo in comparison to rice.

Nevertheless, it is noteworthy that the number of cDNAs found for caffeoyl-CoA O-methyltransferase (CCoAOMT) was nearly the same as that of rice genes. This much higher than average ratio could be a result of duplication of genes encoding this particular enzyme in bamboo. It is also possible that the majority of genes for CCoAOMT had high expression levels so that a larger proportion of the cDNA population was captured for sequencing. Either case could support an important role of CCoAOMT in lignin biosynthesis of Moso bamboo. Interestingly, CCoAOMT was identified as key enzyme that determined lignin content in woody trees such as poplar [36]. Furthermore, the repression of CCoAOMT by antisense led to significant reduction of lignin content of transgenic popular but did not affect normal growth of the transgenic plants [36]. These findings together suggested that CCoAOMT may serve as an effective target for genetic manipulation to reduce pollutants generated from bamboo pulping.

### Phylogeny and evolutionary divergence of bamboo from other grasses

The cloning and sequencing of more than ten thousand bamboo putative FL-cDNAs generated the largest set of unlinked nuclear loci to date for phylogenetic analysis of major grass lineages. Even with 43 sequences, the relationship of bamboo was not unambiguously resolved. Different methods of phylogenetic analyses disagreed whether bamboo was more closely related to rice or barley and wheat. While the disagreement was relatively well supported in the respective phylogenies, the branch leading to either grouping was short (Figure 2), suggesting that the three subfamilies, Bambusoideae, Pooideae, and Ehrhartoideae, diverged rapidly from each other, possibly through a process known as adaptive radiation.

In the similar cases where adaptive radiation was involved, lineage sorting posed a major problem to correct reconstruction of the species phylogeny from individual gene sequences, and as a consequence a large number of independent molecular markers were required to resolve the phylogenetic relationships [37]. In the previous phylogenetic studies of the grass family based on only a few chloroplast and nuclear genes, there seemed to have been a consensus that Bambusoideae and Ehrhartoideae were sister groups, i.e., a closer relationship between bamboo and rice [8,38], until a recent study suggesting that Bamboideae and Pooideae (wheat and barley) were more closely related [39]. Our analysis of the 43 putative orthologous genes demonstrates that the relationship among these three subfamilies remains unresolved and this is mostly likely due to rapid diversification of these grasses. The finding raises an intriguing question of what evolutionary forces drove the rapid diversification of bamboo from other grasses and its adaptation to a very different habitat.

Although a direct answer to this question requires long-term research using integrative approaches, some insights may be gained by analyzing the patterns and rates of gene evolution between bamboo and its close relatives. We found that the number of FL-cDNAs with and without homologs in rice, wheat, and barley is nearly equal (Figure 3A). Interestingly, the shared genes encode mostly enzymes, transporters, and structure proteins, whereas genes unique to bamboo encode more frequently binding and regulatory factors. This suggests that regulatory factors evolved more rapidly than enzymes and structural proteins following the divergence of bamboo from other grasses. It has been widely observed that rapid phenotypic evolution of plants at population and species levels was controlled primarily by regulatory genes [40-42]. The characteristics of gene evolution between bamboo and related subfamilies may represent a signature of this evolutionary mechanism at a higher taxonomic level. We thus hypothesize that rapid evolution of regulatory genes could have played an important role during the rapid adaptive diversification of bamboo from other grasses.

For nearly 6,000 bamboo cDNAs with homologs in rice, there are on ly a few pairs that had a *K*_*A*_/*K*_*S *_ratio larger than 1, a threshold measuring positive selection. The lack of gene with high *K*_*A*_/*K*_*S *_values is likely a result of sufficiently long divergence time between bamboo and rice that has obscured the signal of positive selection driving adaptation. When the *K*_*A*_/*K*_*S *_ratio was compared in a finer scale, we found that genes encoding enzymes, transporters, and structural proteins were more abundant in the categories with the *K*_*A*_/*K*_*S *_ratio smaller than 0.2. In contrast, a larger number of genes encoding binding and regulatory factors had the *K*_*A*_/*K*_*S *_ratio between 0.2 and 0.3 (Additional file 14B). This is consistent with the finding when the overall level of divergence was considered, together indicating that regulatory factors not only evolved more rapidly but were more likely to have evolved in response to natural selection driving rapid adaptive divergence of these grasses. It was probably the rapid divergence of regulatory genes that allowed drastic morphological and physiological evolution of bamboos and rapid adaptation to the new forest habitats.

## Conclusion

This study generated the first large collection of bamboo FL-cDNAs and ESTs and the first genomic resource database for bamboos. This is the initial effort to sequence and analyze the genome of Moso bamboo, which has gained increasingly important ecological and economical values. The analyses of yielded putative FL-cDNAs provided the first glance at the structural and functional features of bamboo genomes. Phylogenetic analyses of the bamboo sequences with those of rice, barley, and wheat yielded suggested that bamboo has diverged from these morphologically and physiologically different relatives possibly through an adaptive radiation. Comparative analyses of bamboo cDNAs and rice genes involved in lignin biosynthesis indicated that genes encoding caffeoyl-CoA O-methyltransferase may serve as effective targets for genetic manipulation of lignin content to reduce pollutants resulting from bamboo pulping. The sequences of the FL-cDNAs and ESTs generated in this study will close a critical gap existing in grass comparative genomics and consequently allow the more efficient development of the grass system for evolutionary and functional studies of plant genes and genomes.

As similar study in other plants, we here provided an enriched putative full-length cDNA data collection and corresponding amino acid sequence information as a start of genomic research. More precise transcripts would be confirmed in coming gene cloning and functional research in bamboo.

## Methods

### Plant materials and cDNA library construction

Five cDNA libraries were constructed for *Phyllostachys heterocycla *cv. *pubescens *from shoots just breaking out from ground, shoots reaching a height of ~40 cm, young leaves, and shoots and roots removed from germinating seeds (Additional file 1). For the first three libraries, shoots and leaves were collected in April from plants naturally grown in the Tianmu-Mountain National Nature Reserve in Zhejiang province of China and immediately preserved in liquid nitrogen and then stored at -80°C in the lab until RNA extraction. Shoots were dug out, and non-lignified tissues at the top of shoots with their roots were sampled. From germinating seeds, 1-2 cm shoots and roots were cut and pooled for library construction. Another cDNA library was constructed from leaves of *Bashania fangiana*, collected in May from the Wolong National Nature Reserve in Sichuan province of China. A Cap-Tagging method was used to construct putative full-length enriched cDNA libraries [19].

### Sequencing and assembling FL-cDNA clones

Approximately 50,000 cDNA clones were randomly selected from the five libraries for 5' end single-pass sequencing using BigDye Terminator Cycle sequencing V2.0 Ready Reaction (Applied Biosystems). PHRED and PHRAP [43,44] were used to generate and assemble raw data. Vector sequences were filtered automatically and low-quality bases (quality value <20) were removed. All 5' tagged ESTs were clustered into contigs or singletons using TIGR Gene Indices clustering tools [45,46]. If sequences share >95% similarity over 80 consecutive bases, they were clustered into one contig. All singletons and one randomly chosen clone from each contig were fully sequenced in both directions, with internal sequencing primers designed for clones longer than 1 kb. To minimize sequence errors, all assembled sequences were manually checked. Programs CAP3 and BLAST were then used to filter redundant sequences judged by having 99% or higher identity [47].

### Sequence analysis

ORFs were predicted using the "getorf" program of EMBOSS package [48], with the longest ORF extracted for each FL-cDNA. ORFs longer than 100 amino acids were analyzed further for codon usage and SSR using the CUSP program implemented in EMBOSS and a perl script, MISA http://pgrc.ipk-gatersleben.de/misa/, respectively. For comparison, cDNA data of Arabidopsis and rice were downloaded from public databases PlantGDB http://www.plantgdb.org/AtGDB/ and NCBI ftp://ftp.ncbi.nih.gov, respectively.

To identify putative alternative splicing variants, program CAP3 [47] was first used to filter singletons with default parameters. The remaining sequences assembled to contigs were subjected to a BLASTN analysis against themselves. Those with sequence identity higher than 99% for at least 100 bp were extracted. The GT-AG rule of exon/intron boundary was then applied using program BLAST2 followed by manual check. To identify putative sense-antisense transcript pairs, all sequences were searched against themselves using BLASTN. Those with plus and minus matching pairs that were at least 50 nucleotide long and had less than 2 bp mismatches were recognized.

A database of plant transposable element peptide sequences was retrieved from the GenBank non-redundant protein database using keywords, including transpose, retro, non-LTR, en/spm, ac/ds, gypsy, copia, polyprotein, mutator, and mudr [24,49]. The bamboo FL-cDNA sequences were then searched against this database using BLASTx with E-value lower than 1e-20.

All bamboo putative full-length cDNA sequences were searched against NCBI nrDB using BLASTX (E-value < 1e-10). The InterPro database was used for identifying putative protein domains [50]. Functional classification followed PFAM [51] and GO terms attached to the InterPro domain names.

### Comparative and phylogenetic analyses with other grasses

Similarity searches were performed with BLASTn and tBLASTx (version 2.2.14) [52] against the public database TIGR Plant Transcript Assemblies http://plantta.tigr.org/; [53]) (including *Oryza sativa, Triticum aestivum, Zea mays, Zea mays B73, Hordeum vulgare, Sorghum bicolor, Saccharum officinarum, Brachypodium distachyon*, and *Panicum virgatum*, and *Arabidopsis thaliana*), with an E-value cutoff of 10^-10 ^(BLASTn) and 10^-6 ^(tBLASTx), respectively. The bamboo cDNAs were also compared with currently available genome sequences of Arabidopsis http://www.plantgdb.org/AtGDB/, rice (IRGSP version 4.0: http://rgp.dna.affrc.go.jp/IRGSP/, *Sorghum bicolor *http://www.gramene.org/Sorghum_bicolor/index.html, and *Populus trichocarpa *http://www.ornl.gov/sci/ipgc/ with BLASTn and tBLASTn, with the E-value cutoff of 10^-1 ^(BLASTn) and 10^-7 ^(tBLASTn), respectively. Finally, the bamboo cDNAs were searched against NCBI ntDB ftp://ftp.ncbi.nih.gov using BLASTN (E-value < 1e-10). For nucleotide similarity search, homologs were recognized as with >75% sequence identity for >50% bamboo FL-cDNA sequences. For tBLASTn and tBLASTx search of amino acid sequences, homologs were recognized as with >60% sequence identity and for >50 consecutive amino acids.

To identify putative orthologs for phylogenetic analyses, we applied a stringent criterion requiring that there was only one homolog identified in any other species when a bamboo cDNA was used as the query in the BLAST search and vice versa. Identification of orthologs in other species was carried out in three steps. 1) Three hits with the best E-value in BLAST were selected. Their DNA sequences were re retrieved from public database by their accession number. 2) The selected sequences were put into ORF prediction again because we found some of them do not carry entire ORF. 3) The predicted amino acid sequences were aligned to the corresponding sequence from bamboo. Only ONE sequence with the highest identity in alignment was considered as a putative ortholog.

For phylogenetic analysis, DNA sequences were aligned using program ClustalW with the default settings [54]. Phylogenetic trees were inferred by maximum likelihood (ML), Neighbour joining (NJ), and Bayesian inference (BI). ML and NJ were implemented with PAUP 4.0b10 [55] and the branch-and-bound algorithm was used for tree searching. BI was employed for phylogenetic reconstruction using the MrBayes software of version 3.1 [56]. For sequence divergence, synonymous (*K*_*S*_) and nonsynonymous (*K*_*A*_) distances were estimated using program PAL2NAL [57].

### Analysis of EST clusters for gene/genome duplication

ESTs of Moso bamboo were aligned with the putative FL-cDNAs using the ungapped BLASTN search at the highest E-value of 0.0. For those FL-cDNAs with at least six EST counter parts, the corresponding ESTs were aligned with CLUSTAL 2.0.11 http://software.informer.com/getfree-clustalx2-cite/; [58]). For the resulting EST clusters, we retained those with any given sequence polymorphism supported by at least two members of the ESTs to avoid artifacts of sequence errors. Only those EST clusters that covered the entire predicted coding regions of the FL-cDNA were sampled for estimating sequence divergence. Synonymous substitutions (*K*_*S*_) between homologs were calculated [59,60]. Divergence time were estimated based on T = *K*_*S*_/2*r *using the divergence rate *r *= 6.5 × 10^-9 ^mutations per synonymous site per year [16].

### Databasing ESTs and FL-cDNAs of Moso bamboo

To facilitate the access and utilization of putative FL-cDNA and EST sequences generated for Moso bamboo, we developed a database to store and search these sequences and their annotations. The web interface of this database was constructed in PHP script and the data were stored in a relational database management system, MYSQL. Search function and display were built with a combination of SQL commands and PHP script.

Sequence data from this article can be found in the GenBank under accession numbers FP091249-FP101855.

## Authors' contributions

ZJ, ZP, and BH designed the project. LL, XL, and ZG prepared plant materials. XL, T H, and XY constructed cDNA libraries. QF, JG, QW, and DF conducted DNA sequencing. TL, YL, CZ, and BH analyzed data. TL, BH, ZP, and ZJ wrote the paper. All authors read and approved the final manuscript.

## Supplementary Material

Additional file 1 **Forest, habitat, and morphology of Moso bamboo**. Upper left, bamboo forest in South area (Yibin City) of Sichuan Province, China. Lower left and upper right, mature individuals. Lower right, young shoots. Click here for file

Additional file 2 **Comparison of GC content in 5'-UTR, coding, and 3'-UTR regions among rice, Arabidopsis, and bamboo**. Figure **A**, **B**, and **C **show the GC content in 5'-UTR, coding, and 3'-UTR regions, respectively. The red curve exhibits the frequency for that of Arabidopsis, green for rice, and blue for bamboo. Click here for file

Additional file 3 **Codon usage estimated from bamboo, rice, and Arabidopsis FL-cDNAs**. Click here for file

Additional file 4 **Putative alternative splicing**. **A**. Illustration of alternative splicing types. Exons are represented by boxes and introns by lines. Constitutive exons are shown in gray. **B**. Relative frequency of putative alternative splicing types of bamboo cDNAs. Click here for file

Additional file 5 **Sense-antisense pairs found in bamboo FL-cDNAs**. Click here for file

Additional file 6 **Top 20 most common protein families identified from bamboo FL-cDNAs according to PFAM database**. Click here for file

Additional file 7 **Top 10 most abundant transcription factors found in bamboo FL-cDNAs**. Click here for file

Additional file 8 **GO molecular functions of bamboo FL-cDNAs**. Click here for file

Additional file 9 **Phylogeny of bamboo and rice genes encoding nine key enzymes in the lignin biosythesis pathway**. Phylogeny of each gene was inferred from Neighbor Joining method. Each sequence is named by its GenBank accession number. Asterisks indicate bamboo cDNAs. Scales measuring branch length of corresponding gene trees indicate 10 nucleotide substitutions. Click here for file

Additional file 10 **Putative homologs of bamboo cDNA identified in TIGR Plant Transcript Assemblies dataset**. Click here for file

Additional file 11 **Putative homologs of bamboo cDNA identified in genome sequences of other plants**. Click here for file

Additional file 12 **43 groups of putative orthologs identified among bamboo, rice, maize, wheat, Sorghum, Sugarcane, barley, Brachypodium, switchgrass, and Arabidopsis**. Click here for file

Additional file 13 **Distribution of *K*_*A*_, *K*_*S*_, and *K*_*A*_*/K*_*S *_of bamboo cDNAs according to their divergence with rice cDNAs**. **A**. Distribution of KA and KS of rice-bamboo ortholog pairs. Red line represented that cDNAs are arranged with synonymous divergence. Blue dots indicated corresponding nonsynonymous divergence. **B**. Distribution of and of *K*_*S *_and *K*_*A*_*/K*_*S*_. Click here for file

Additional file 14 **Predicted chromosomal distribution and functional classification of cDNAs with different rice-bamboo divergence**. (A) Distribution of rice cDNAs on chromosomes. Red represents cDNAs with *K*_*S *_< 0.375 and blue represents cDNAs with 0.375 <*K*_*S *_< 0.75, in comparison with bamboo. We compared chromosomal distribution of rice homologs that diverged at rates between *K*_*S *_< 0.375 and 0.375 <*K*_*S *_< 0.75. For every 5 Mb interval of rice chromosomes, the number of genes between the two categories are significantly correlated (*r*^2 ^= 0.70, *P *< 0.001), indicating that chromosomal location did not affect *K*_*S *_which represents neutral evolutionary rates. Furthermore, we tested whether the rice homologs of the bamboo cDNAs are randomly distributed on rice chromosomes. We found that for every 5 Mb the distribution of the homologs were significantly correlated with that of all rice genes (*r*^2 ^= 0.25, *P *< 0.001), suggesting bamboo genes isolated in this study are likely to have been sampled randomly across the genome (data not shown). (B) Functional classification of cDNAs with *K*_*A*_/*K*_*S *_< 0.1, between 0.1 and 0.2, and between 0.2 and 0.3. We compared the functional classification of sequence pairs with *K*_*A*_/*K*_*S *_in the following intervals: 0 - 0.1, 0.1 - 0.2, and 0.2 - 0.3. This accounted for the vast majority of sequence pairs and partitioned them into the following categories: 1,445 pairs, 38.5% (0.0-0.1), 1,434 pairs, 38.2% (0.1-0.2), and 595 pairs, 15.8% (0.2-0.3). While there is no clear difference in frequency distribution of the functional classification for more than half of gene types, sequence pairs with the lowest *K*_*A*_/*K*_*S *_values are more abundant in structural molecules whereas sequences with the highest *K*_*A*_/*K*_*S *_values are more abundant in regulators. Click here for file

Additional file 15 **Homepage of the Moso bamboo cDNA database on website**. The database and source code are available at http://www.ncgr.ac.cn/mbcd/. Click here for file
