# Supplementary figures and images for: Genome-wide characterization of the biggest grass, bamboo, based on 10,608 putative full-length cDNA sequences
Source: BMC Plant Biol. 2010 Jun 18;10:116. doi: 10.1186/1471-2229-10-116 (PMC3017805; doi:10.1186/1471-2229-10-116)

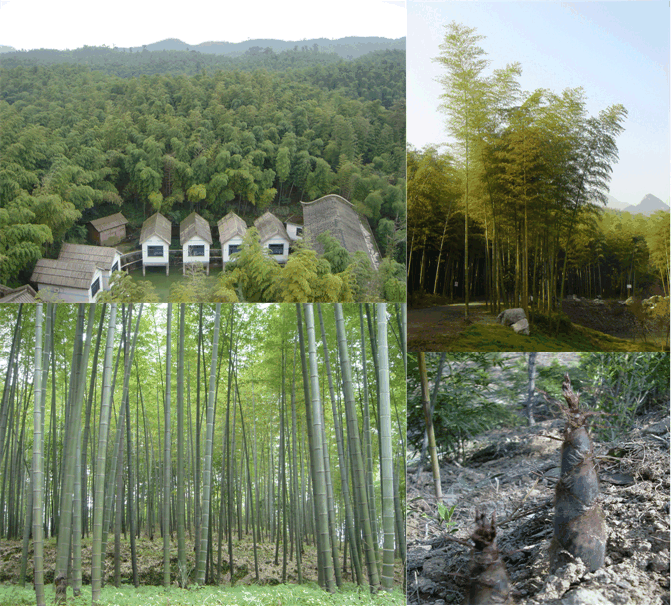

Supplement: Additional file 1 — Forest, habitat, and morphology of Moso bamboo. Upper left, bamboo forest in South area (Yibin City) of Sichuan Province, China. Lower left and upper right, mature individuals. Lower right, young shoots. [file 1471-2229-10-116-S1.GIF]

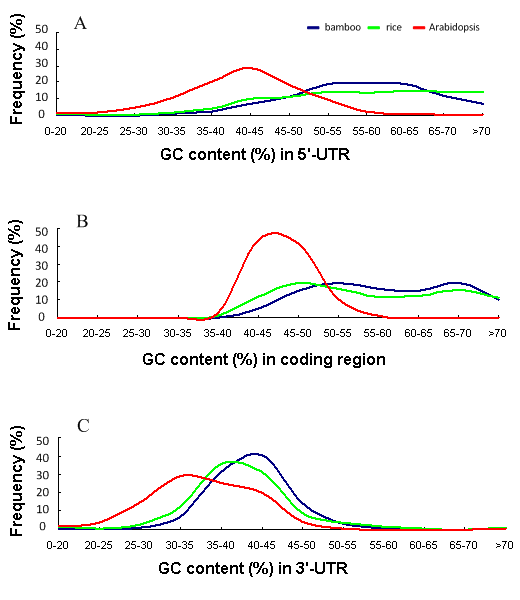

Supplement: Additional file 2 — Comparison of GC content in 5'-UTR, coding, and 3'-UTR regions among rice, Arabidopsis, and bamboo. Figure A, B, and C show the GC content in 5'-UTR, coding, and 3'-UTR regions, respectively. The red curve exhibits the frequency for that of Arabidopsis, green for rice, and blue for bamboo. [file 1471-2229-10-116-S2.GIF]

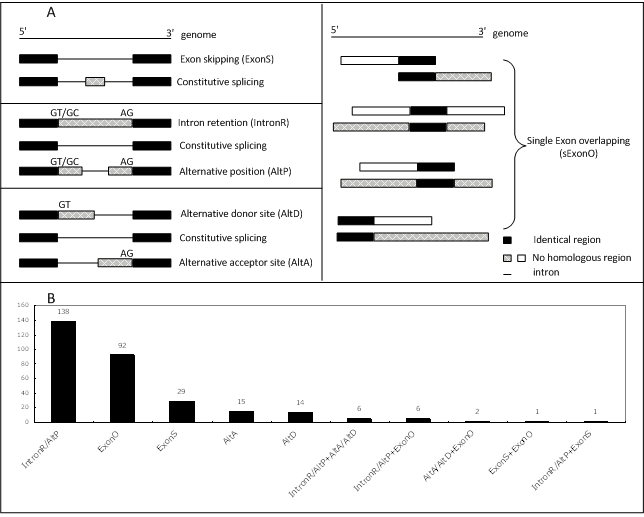

Supplement: Additional file 4 — Putative alternative splicing. A. Illustration of alternative splicing types. Exons are represented by boxes and introns by lines. Constitutive exons are shown in gray. B. Relative frequency of putative alternative splicing types of bamboo cDNAs. [file 1471-2229-10-116-S4.GIF]

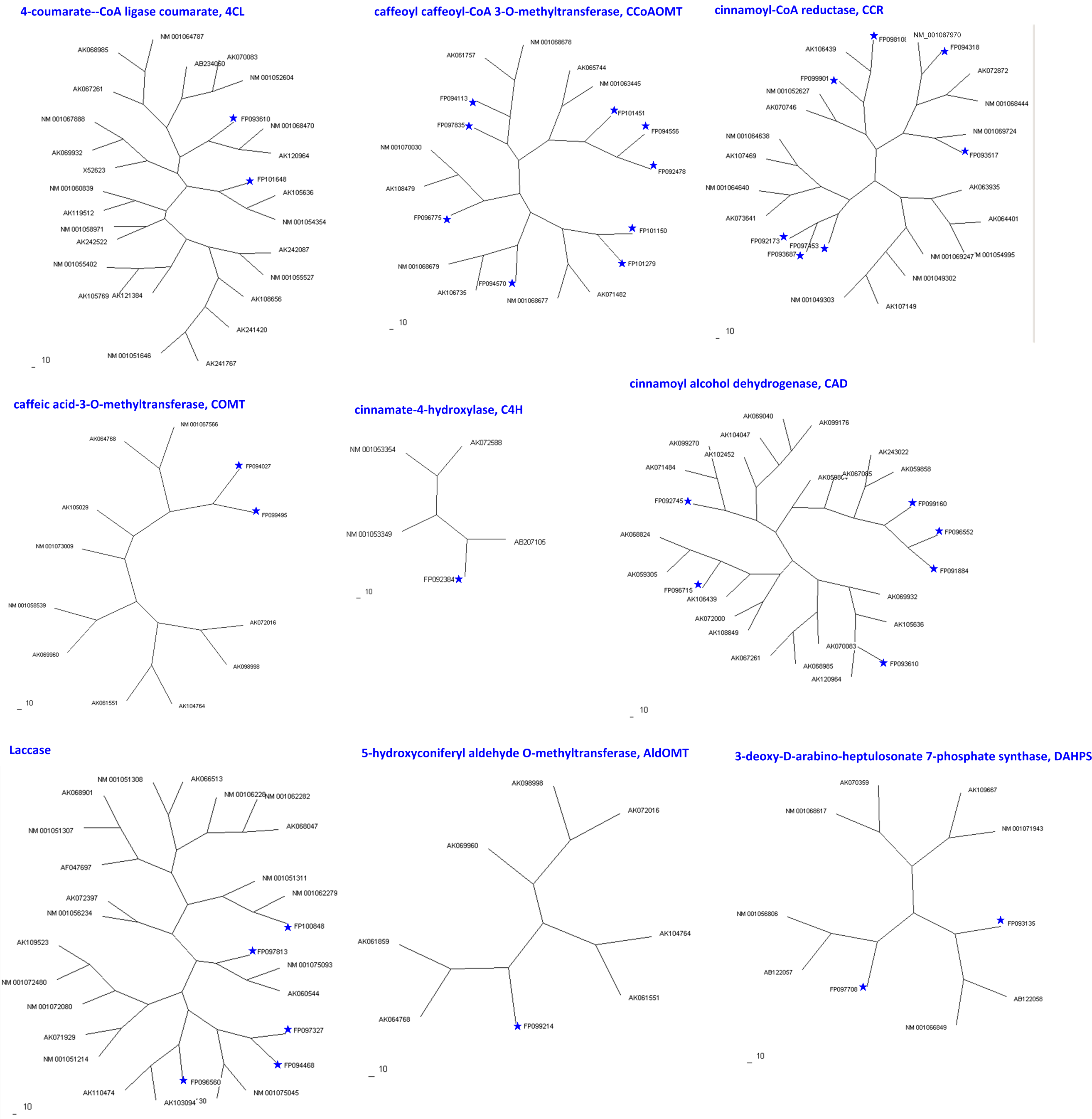

Supplement: Additional file 9 — Phylogeny of bamboo and rice genes encoding nine key enzymes in the lignin biosythesis pathway. Phylogeny of each gene was inferred from Neighbor Joining method. Each sequence is named by its GenBank accession number. Asterisks indicate bamboo cDNAs. Scales measuring branch length of corresponding gene trees indicate 10 nucleotide substitutions. [file 1471-2229-10-116-S9.GIF]

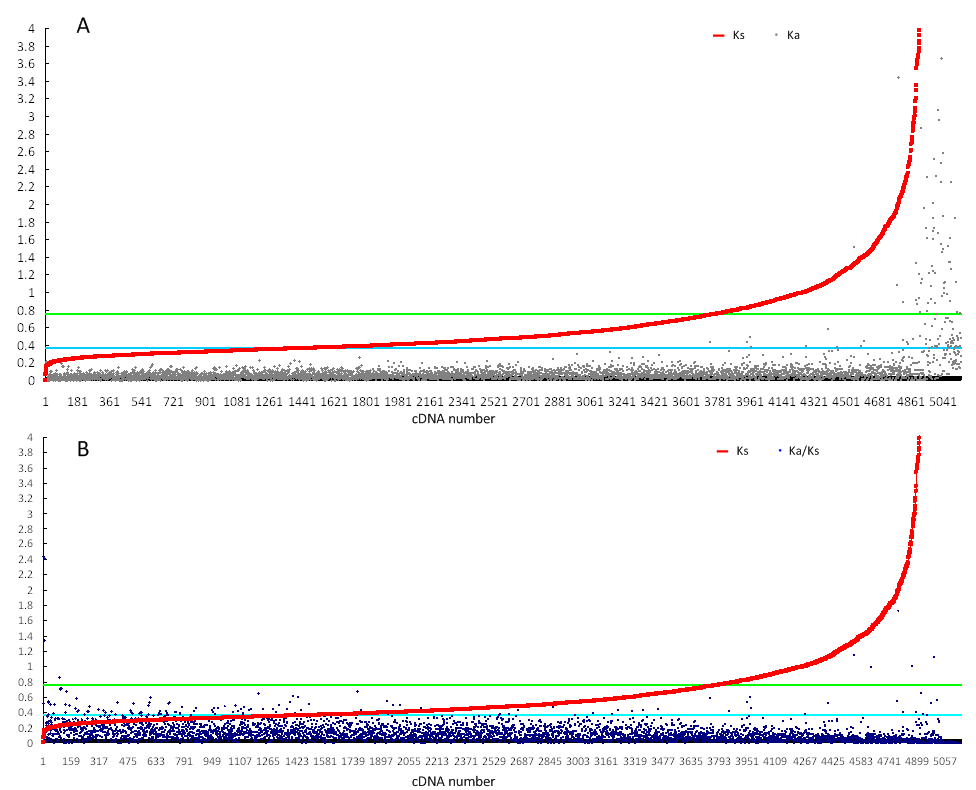

Supplement: Additional file 13 — Distribution of KA, KS, and KA/KS of bamboo cDNAs according to their divergence with rice cDNAs. A. Distribution of KA and KS of rice-bamboo ortholog pairs. Red line represented that cDNAs are arranged with synonymous divergence. Blue dots indicated corresponding nonsynonymous divergence. B. Distribution of and of KS and KA/KS. [file 1471-2229-10-116-S13.GIF]

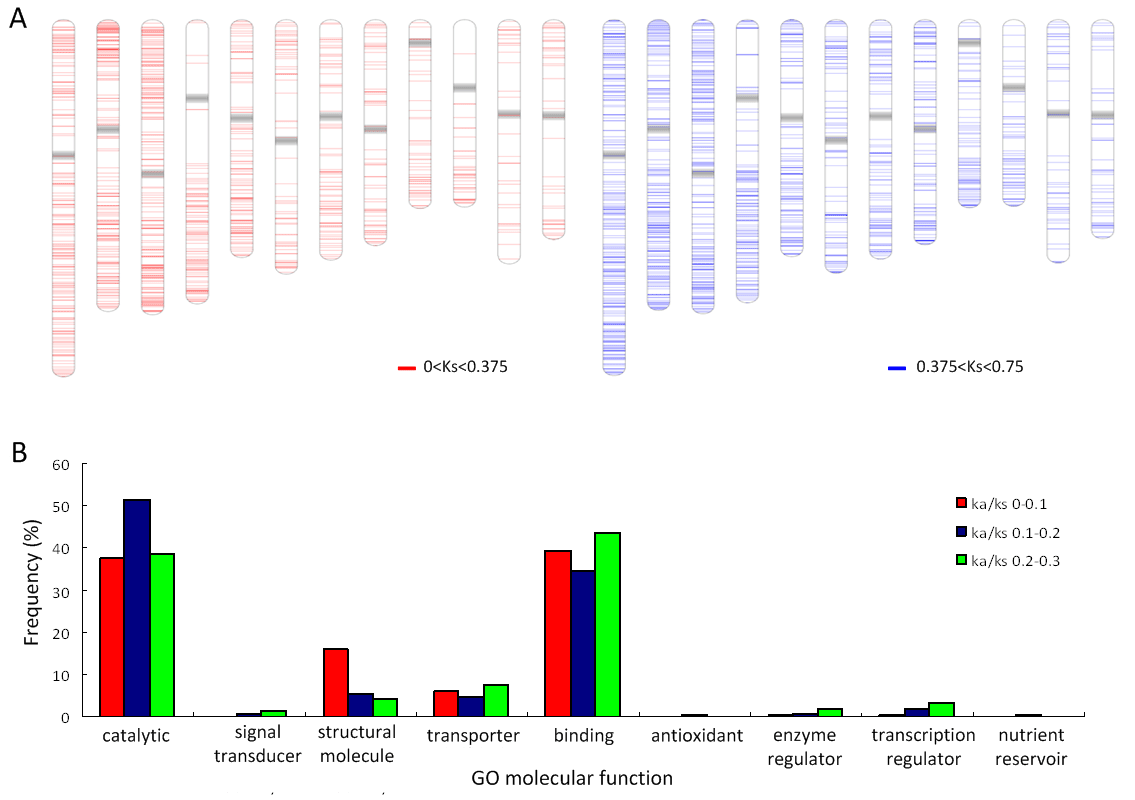

Supplement: Additional file 14 — Predicted chromosomal distribution and functional classification of cDNAs with different rice-bamboo divergence. (A) Distribution of rice cDNAs on chromosomes. Red represents cDNAs with KS < 0.375 and blue represents cDNAs with 0.375 <KS < 0.75, in comparison with bamboo. We compared chromosomal distribution of rice homologs that diverged at rates between KS < 0.375 and 0.375 <KS < 0.75. For every 5 Mb interval of rice chromosomes, the number of genes between the two categories are significantly correlated (r2 = 0.70, P < 0.001), indicating that chromosomal location did not affect KS which represents neutral evolutionary rates. Furthermore, we tested whether the rice homologs of the bamboo cDNAs are randomly distributed on rice chromosomes. We found that for every 5 Mb the distribution of the homologs were significantly correlated with that of all rice genes (r2 = 0.25, P < 0.001), suggesting bamboo genes isolated in this study are likely to have been sampled randomly across the genome (data not shown). (B) Functional classification of cDNAs with KA/KS < 0.1, between 0.1 and 0.2, and between 0.2 and 0.3. We compared the functional classification of sequence pairs with KA/KS in the following intervals: 0 - 0.1, 0.1 - 0.2, and 0.2 - 0.3. This accounted for the vast majority of sequence pairs and partitioned them into the following categories: 1,445 pairs, 38.5% (0.0-0.1), 1,434 pairs, 38.2% (0.1-0.2), and 595 pairs, 15.8% (0.2-0.3). While there is no clear difference in frequency distribution of the functional classification for more than half of gene types, sequence pairs with the lowest KA/KS values are more abundant in structural molecules whereas sequences with the highest KA/KS values are more abundant in regulators. [file 1471-2229-10-116-S14.GIF]

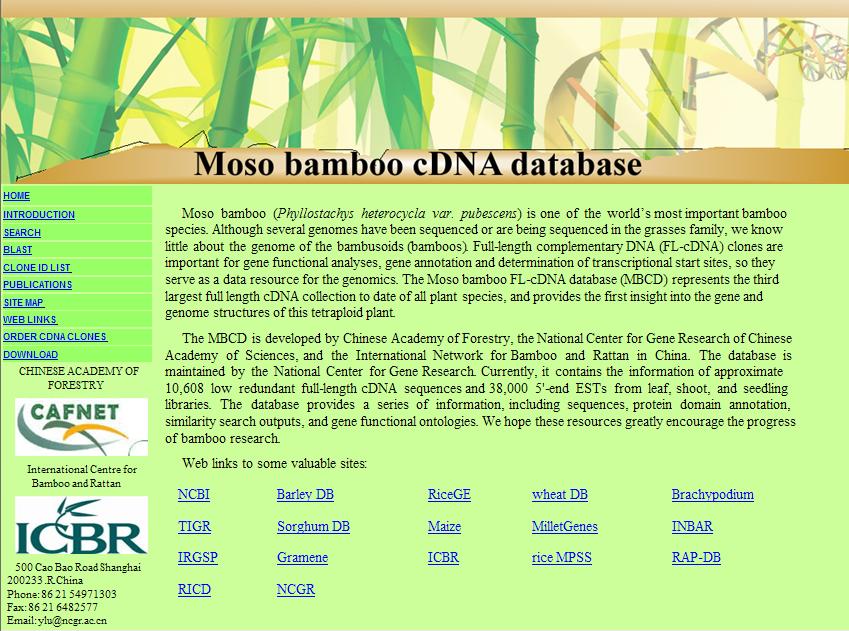

Supplement: Additional file 15 — Homepage of the Moso bamboo cDNA database on website. The database and source code are available at http://www.ncgr.ac.cn/mbcd/. [file 1471-2229-10-116-S15.JPEG]
