# Supplementary material for: Genome-wide characterization of the biggest grass, bamboo, based on 10,608 putative full-length cDNA sequences
Source: BMC Plant Biol. 2010 Jun 18;10:116. doi: 10.1186/1471-2229-10-116 (PMC3017805; doi:10.1186/1471-2229-10-116)
Supplement: Additional file 3 — Codon usage estimated from bamboo, rice, and Arabidopsis FL-cDNAs. [file 1471-2229-10-116-S3.DOC]

**Additional file 3.** Codon usage estimated from bamboo, rice, and Arabidopsis FL-cDNAs.

| Codon | Amino Acid | Fraction* | | |
| --- | --- | --- | --- | --- |
|  |  | Bamboo | Rice | Arabidopsis |
| GCA | A | 0.15 | 0.19 | 0.25 |
| GCC | A | 0.37 | 0.33 | 0.17 |
| GCG | A | 0.28 | 0.27 | 0.14 |
| GCT | A | 0.19 | 0.22 | 0.44 |
| TGC | C | 0.77 | 0.69 | 0.20 |
| TGT | C | 0.23 | 0.32 | 0.58 |
| GAC | D | 0.62 | 0.54 | 0.33 |
| GAT | D | 0.38 | 0.46 | 0.67 |
| GAA | E | 0.27 | 0.34 | 0.50 |
| GAG | E | 0.73 | 0.66 | 0.50 |
| TTC | F | 0.72 | 0.65 | 0.51 |
| TTT | F | 0.28 | 0.35 | 0.49 |
| GGA | G | 0.17 | 0.19 | 0.36 |
| GGC | G | 0.44 | 0.40 | 0.15 |
| GGG | G | 0.22 | 0.21 | 0.15 |
| GGT | G | 0.18 | 0.20 | 0.34 |
| CAC | H | 0.66 | 0.56 | 0.41 |
| CAT | H | 0.34 | 0.44 | 0.59 |
| ATA | I | 0.16 | 0.19 | 0.23 |
| ATC | I | 0.56 | 0.48 | 0.37 |
| ATT | I | 0.28 | 0.33 | 0.40 |
| AAA | K | 0.22 | 0.30 | 0.48 |
| AAG | K | 0.78 | 0.70 | 0.52 |
| CTA | L | 0.06 | 0.07 | 0.10 |
| CTC | L | 0.36 | 0.30 | 0.19 |
| CTG | L | 0.27 | 0.25 | 0.11 |
| CTT | L | 0.15 | 0.17 | 0.26 |
| TTA | L | 0.04 | 0.06 | 0.13 |
| TTG | L | 0.13 | 0.15 | 0.22 |
| ATG | M | 1.00 | 1.00 | 1.00 |
| AAC | N | 0.66 | 0.57 | 0.50 |
| AAT | N | 0.34 | 0.43 | 0.50 |
| CCA | P | 0.20 | 0.25 | 0.32 |
| CCC | P | 0.27 | 0.22 | 0.12 |
| CCG | P | 0.32 | 0.30 | 0.19 |
| CCT | P | 0.20 | 0.24 | 0.38 |
| CAA | Q | 0.29 | 0.35 | 0.55 |
| CAG | Q | 0.71 | 0.65 | 0.46 |
| AGA | R | 0.12 | 0.15 | 0.34 |
| AGG | R | 0.24 | 0.24 | 0.19 |
| CGA | R | 0.06 | 0.08 | 0.12 |
| CGC | R | 0.30 | 0.26 | 0.08 |
| CGG | R | 0.19 | 0.17 | 0.09 |
| CGT | R | 0.09 | 0.10 | 0.17 |
| AGC | S | 0.22 | 0.20 | 0.13 |
| AGT | S | 0.08 | 0.11 | 0.14 |
| TCA | S | 0.12 | 0.16 | 0.20 |
| TCC | S | 0.27 | 0.21 | 0.14 |
| TCG | S | 0.16 | 0.16 | 0.11 |
| TCT | S | 0.14 | 0.16 | 0.29 |
| ACA | T | 0.18 | 0.24 | 0.29 |
| ACC | T | 0.39 | 0.32 | 0.21 |
| ACG | T | 0.24 | 0.23 | 0.16 |
| ACT | T | 0.19 | 0.22 | 0.34 |
| GTA | V | 0.08 | 0.10 | 0.14 |
| GTC | V | 0.34 | 0.31 | 0.20 |
| GTG | V | 0.38 | 0.36 | 0.25 |
| GTT | V | 0.20 | 0.23 | 0.41 |
| TGG | W | 1.00 | 1.00 | 1.00 |
| TAC | Y | 0.72 | 0.63 | 0.50 |
| TAT | Y | 0.28 | 0.37 | 0.50 |
| TAA | stop | 0.27 | 0.25 | 0.36 |
| TGA | stop | 0.44 | 0.46 | 0.44 |
| TAG | stop | 0.28 | 0.27 | 0.20 |

*The proportion of usage of a given codon among its redundant set.
