# Supplementary material for: Genome-wide characterization of the biggest grass, bamboo, based on 10,608 putative full-length cDNA sequences
Source: BMC Plant Biol. 2010 Jun 18;10:116. doi: 10.1186/1471-2229-10-116 (PMC3017805; doi:10.1186/1471-2229-10-116)
Supplement: Additional file 5 — Sense-antisense pairs found in bamboo FL-cDNAs. [file 1471-2229-10-116-S5.DOC]

**Additional file 5.** Sense-antisense pairs found in bamboo FL-cDNAs. A and B represent sense and antisense pairs, respectively.

| No. | A | Length (bp) | Protein | B | Length (bp) | Protein | Identity (%) | A-hit-start | A-hit-end | B-hit-start | B-hit-end |
| --- | --- | --- | --- | --- | --- | --- | --- | --- | --- | --- | --- |
| 1 | FP091351 | 1386 | putative | FP092217 | 705 | pistarvation-induced | 100 | 699 | 771 | 80 | 8 |
| 2 | FP092840 | 1457 | hypothetical | FP092376 | 2247 | hypothetical | 99 | 1 | 698 | 2083 | 1386 |
| 3 | FP094666 | 1319 | No | FP092944 | 1994 | No | 100 | 1 | 439 | 1132 | 694 |
| 4 | FP093005 | 1165 | hypothetical | FP095602 | 1209 | hypothetical | 99 | 1 | 960 | 960 | 1 |
| 5 | FP096002 | 1447 | No | FP093252 | 946 | No | 99 | 365 | 1029 | 665 | 1 |
| 6 | FP099084 | 1203 | putative | FP093663 | 1231 | hypothetical | 100 | 233 | 561 | 397 | 69 |
| 7 | FP100239 | 1355 | putative | FP098896 | 934 | putative | 99 | 1 | 235 | 732 | 498 |
| 8 | FP093512 | 511 | putative | FP092242 | 781 | putative | 99 | 109 | 1042 | 934 | 1 |
| 9 | FP094381 | 665 | No | FP093756 | 1723 | No | 100 | 225 | 398 | 554 | 381 |
| 10 | FP097603 | 741 | No | FP095881 | 1398 | hypothetical | 100 | 1 | 402 | 1050 | 649 |
| 11 | FP097817 | 737 | No | FP094299 | 787 | No | 100 | 589 | 741 | 1258 | 1106 |
| 12 | FP097547 | 784 | No | FP096881 | 1850 | No | 100 | 17 | 527 | 511 | 1 |
|  | FP096008 | 782 | putative |  |  |  | 100 | 1 | 514 | 1237 | 724 |
