# Supplementary material for: Genome-wide characterization of the biggest grass, bamboo, based on 10,608 putative full-length cDNA sequences
Source: BMC Plant Biol. 2010 Jun 18;10:116. doi: 10.1186/1471-2229-10-116 (PMC3017805; doi:10.1186/1471-2229-10-116)
Supplement: Additional file 6 — Top 20 most common protein families identified from bamboo FL-cDNAs according to PFAM database. [file 1471-2229-10-116-S6.DOC]

**Additional file 6.** Top 20 most common protein families identified from bamboo FL-cDNAs according to PFAM database.

| PFAM No. | Description | Number |
| --- | --- | --- |
| PF00125 | Histone | 117 |
| PF00076 | RNA recognition motif | 107 |
| PF00069 | Protein kinase | 86 |
| PF00141 | Plant peroxidase | 81 |
| PF00179 | Ubiquitin-conjugating enzyme | 81 |
| PF02798 | Glutathione S-transferase | 78 |
| PF00234 | Plant lipid transfer protein/seed storage/trypsin-alpha amylase inhibitor | 78 |
| PF00071 | Ras | 75 |
| PF00190 | Cupin 1 | 64 |
| PF00847 | Pathogenesis-related transcriptional factor and ERF | 63 |
| PF00230 | Major intrinsic protein | 53 |
| PF00504 | Chlorophyll A-B binding protein | 50 |
| PF00657 | Lipolytic enzyme, G-D-S-L | 41 |
| PF00227 | 20S proteasome, A and B subunits | 41 |
| PF00025 | ARF/SAR superfamily | 39 |
| PF02365 | No apical meristem (NAM) protein | 39 |
| PF03106 | DNA-binding WRKY | 31 |
| PF02298 | Plastocyanin-like | 30 |
| PF00067 | Cytochrome P450 | 30 |
| PF00240 | Ubiquitin | 30 |
