# Supplementary material for: Genome-wide characterization of the biggest grass, bamboo, based on 10,608 putative full-length cDNA sequences
Source: BMC Plant Biol. 2010 Jun 18;10:116. doi: 10.1186/1471-2229-10-116 (PMC3017805; doi:10.1186/1471-2229-10-116)
Supplement: Additional file 7 — Top 10 most abundant transcription factors found in bamboo FL-cDNAs. [file 1471-2229-10-116-S7.DOC]

**Additional file 7.** Top 10 most abundant transcription factors found in bamboo FL-cDNAs.

| Category of domain | Number |
| --- | --- |
| ERF | 71 |
| Myb | 41 |
| Zinc finger (Including C2H2, CCCH, GATA, PHD and LIM) | 39 |
| WRKY | 37 |
| Homeobox | 33 |
| bZIP | 27 |
| bHLH | 22 |
| NAC | 22 |
| CBF/NF-Y/archaeal histone | 15 |
| MADS | 12 |
