# Supplementary material for: Genome-wide characterization of the biggest grass, bamboo, based on 10,608 putative full-length cDNA sequences
Source: BMC Plant Biol. 2010 Jun 18;10:116. doi: 10.1186/1471-2229-10-116 (PMC3017805; doi:10.1186/1471-2229-10-116)
Supplement: Additional file 8 — GO molecular functions of bamboo FL-cDNAs. [file 1471-2229-10-116-S8.DOC]

**Additional file 8.** GO molecular functions of bamboo FL-cDNAs.

| Category | Number | Percentage |
| --- | --- | --- |
| Binding | 3792 | 46.28 |
| Catalytic activity | 2879 | 35.14 |
| Structural molecule activity | 480 | 5.86 |
| Transporter activity | 429 | 5.24 |
| Transcription regulator activity | 298 | 3.64 |
| Antioxidant activity | 103 | 1.26 |
| Nutrient reservoir activity | 72 | 0.88 |
| Translation regulator activity | 61 | 0.74 |
| Enzyme regulator activity | 46 | 0.56 |
| Signal transducer activity | 33 | 0.40 |
| Motor activity | 1 | 0.01 |
|  |  |  |
