# Supplementary material for: Genome-wide characterization of the biggest grass, bamboo, based on 10,608 putative full-length cDNA sequences
Source: BMC Plant Biol. 2010 Jun 18;10:116. doi: 10.1186/1471-2229-10-116 (PMC3017805; doi:10.1186/1471-2229-10-116)
Supplement: Additional file 10 — Putative homologs of bamboo cDNA identified in TIGR Plant Transcript Assemblies dataset. [file 1471-2229-10-116-S10.DOC]

**Additional file 10.** Putative homologs of bamboo cDNA identified in TIGR Plant Transcript Assemblies dataset.

| Species | No. sequences in database | blastn; E-value<1e-10; identity>75% and overall bamboo cDNA length >50% matched to others | | tblastx; E-value<1e-6; identity>60%; length of matched continuous reads >50 aa | |
| --- | --- | --- | --- | --- | --- |
|  |  | No. bamboo hits | Percentage (%) | No. bamboo hits | Percentage (%) |
| *Oryza sativa* | 247,516 | 5,216 | 49.2 | 8,652 | 81.2 |
| *Triticum aestivum* | 319,949 | 4,157 | 39.2 | 8,361 | 78.8 |
| *Zea mays* | 284,332 | 4,040 | 38.1 | 8,324 | 78.5 |
| *Zea mays B73* | 204,496 | 3,815 | 36.0 | 8,169 | 77.0 |
| *Hordeum vulgare* | 123,351 | 3,795 | 35.8 | 8,095 | 76.3 |
| *Sorghum bicolor* | 48,932 | 3,272 | 30.8 | 7,642 | 72.0 |
| *Saccharum officinarum* | 157,190 | 2,966 | 28.0 | 7,995 | 75.3 |
| *Brachypodium distachyon* | 11,144 | 2,173 | 20.5 | 5,674 | 53.5 |
| *Panicum virgatum* | 7,638 | 1,374 | 13.0 | 4,603 | 43.4 |
| *Arabidopsis thaliana* | 148,368 | 329 | 3.1 | 6,159 | 58.1 |
