# Supplementary material for: Genome-wide characterization of the biggest grass, bamboo, based on 10,608 putative full-length cDNA sequences
Source: BMC Plant Biol. 2010 Jun 18;10:116. doi: 10.1186/1471-2229-10-116 (PMC3017805; doi:10.1186/1471-2229-10-116)
Supplement: Additional file 11 — Putative homologs of bamboo cDNA identified in genome sequences of other plants. [file 1471-2229-10-116-S11.DOC]

**Additional file 11.** Putative homologs of bamboo cDNA identified in genome sequences of other plants.

| Species | blastn; E-value<1e-1; identity>75% and overall bamboo cDNA length >50% matched to genome sequences | | tblastn; E-value<1e-7; identity>60%; length of matched continuous reads >50 aa | |
| --- | --- | --- | --- | --- |
|  | No. bamboo hits | Percentage (%) | No. bamboo hits | Percentage (%) |
| *Oryza sativa* | 4,418 | 41.6 | 6,876 | 64.8 |
| *Sorghum bicolor* | 3,490 | 32.9 | 6,703 | 63.2 |
| *Populus trichocarpa* | 157 | 1.5 | 3,829 | 36.1 |
| *Arabidopsis thaliana* | 137 | 1.3 | 3,858 | 36.4 |
