# Supplementary material for: Genome-wide characterization of the biggest grass, bamboo, based on 10,608 putative full-length cDNA sequences
Source: BMC Plant Biol. 2010 Jun 18;10:116. doi: 10.1186/1471-2229-10-116 (PMC3017805; doi:10.1186/1471-2229-10-116)
Supplement: Additional file 12 — 43 groups of putative orthologs identified among bamboo, rice, maize, wheat, Sorghum, Sugarcane, barley, Brachypodium, switchgrass, and Arabidopsis. [file 1471-2229-10-116-S12.DOC]

**Additional file 12.** 43 groups of putative orthologs identified among bamboo, rice, maize, wheat, Sorghum, Sugarcane, barley, Brachypodium, switchgrass, and Arabidopsis.

| No. | Protein descriptions | *Phyllostachys*  *pubescens* | *Oryza sativa* | *Zea mays* | *Zea mays B73* | *Triticum aestivum* | *Sorghum bicolor* | *Saccharum officinarum* | *Hordeum vulgare* | *Brachypodium distachyon* | *Panicum virgatum* | *Arabidopsis* |
| --- | --- | --- | --- | --- | --- | --- | --- | --- | --- | --- | --- | --- |
|  |  | Accession No. | TIGR-TA-ID | TIGR-TA-ID | TIGR-TA-ID | TIGR-TA-ID | TIGR-TA-ID | TIGR-TA-ID | TIGR-TA-ID | TIGR-TA-ID | TIGR-TA-ID | TIGR-TA-ID |
| 1 | alpha tubulin | FP100535 | TA33668_4530 | TA95860_4577 | TA1818_4577999 | TA55287_4565 | TA21349_4558 | TA23872_4547 | TA29885_4513 | TA66_15368 | TA1763_38727 | TA28440_3702 |
| 2 | ATP sulfurylase | FP094761 | TA41501_4530 | TA111574_4577 | TA10437_4577999 | TA68382_4565 | TA23792_4558 | TA32137_4547 | TA31152_4513 | TA304_15368 | DN141459 | TA40405_3702 |
| 3 | DnaJ like protein | FP100787 | TA37087_4530 | AY103727 | TA4395_4577999 | TA53255_4565 | TA20995_4558 | TA26011_4547 | TA28157_4513 | TA421_15368 | TA2766_38727 | TA26057_3702 |
| 4 | ubiquitin conjugating enzyme E2 | FP099182 | TA40731_4530 | DR785970 | DR785970 | CK216668 | CD226737 | CA289095 | BU991349 | DV486849 | TA2962_38727 | BE038411 |
| 5 | chloroplast light-harvesting chlorophyll a/b binding protein | FP097278 | CX109006 | TA100993_4577 | TA1847_4577999 | TA56359_4565 | TA21271_4558 | TA23764_4547 | TA30041_4513 | DV483707 | TA1713_38727 | TA26755_3702 |
| 6 | Magnesium-protoporphyrin IX monomethyl ester [oxidative] cyclase | FP092998 | TA37877_4530 | TA101769_4577 | TA15800_4577999 | TA63326_4565 | TA20929_4558 | TA32428_4547 | TA31399_4513 | TA103_15368 | TA1986_38727 | TA29470_3702 |
| 7 | 40S ribosomal protein S15A | FP099768 | TA32091_4530 | DW530847 | TA5115_4577999 | CK166611 | BG557777 | CA126612 | TA28225_4513 | TA367_15368 | DN146520 | TA29417_3702 |
| 8 | chloroplastic aldolase | FP098166 | TA33449_4530 | TA101241_4577 | TA3246_4577999 | TA64036_4565 | TA22022_4558 | TA41945_4547 | TA29970_4513 | TA19_15368 | TA2170_38727 | CNS0A43J |
| 9 | 14-3-3-like protein | FP098808 | TA29653_4530 | TA98133_4577 | TA2143_4577999 | TA52757_4565 | TA20677_4558 | TA24465_4547 | TA30325_4513 | TA182_15368 | TA2542_38727 | TA33748_3702 |
| 10 | ethylene-responsive small GTP-binding protein | FP099622 | TA41140_4530 | DT943921 | DT943921 | TA63057_4565 | TA25726_4558 | TA30801_4547 | TA39871_4513 | TA1006_15368 | TA3344_38727 | TA30887_3702 |
| 11 | 60S ribosomal protein L10-1 | FP094942 | CX109297 | CO443088 | TA2208_4577999 | CD939263 | TA21056_4558 | TA32291_4547 | BG309003 | TA524_15368 | TA2006_38727 | TA28109_3702 |
| 12 | histone H3.2 | FP101060 | CX109245 | CO461382 | TA1391_4577999 | CD924111 | TA22144_4558 | CA241200 | BG343702 | TA322_15368 | DN142460 | TA30637_3702 |
| 13 | 60S ribosomal protein L36a/L44 | FP091985 | CX109189 | CF040889 | TA6770_4577999 | CA484775 | TA21532_4558 | CA223011 | BQ665685 | DV472606 | DN141794 | DR321391 |
| 14 | ribosomal protein L15 | FP101592 | TA35872_4530 | TA103857_4577 | TA3605_4577999 | TA62570_4565 | CX606705 | CA133490 | TA28101_4513 | TA1799_15368 | DN152228 | TA26049_3702 |
| 15 | glutathione peroxidase | FP096749 | CK072350 | TA107825_4577 | TA13749_4577999 | DR739963 | TA23817_4558 | CA106470 | BM816591 | DV474217 | DN143025 | TA30591_3702 |
| 16 | putative 40s ribosomal protein S23 | FP093948 | CX102515 | DN212037 | DN212037 | CK214146 | BI074397 | CA218551 | TA31531_4513 | DV472620 | DN152155 | BE038383 |
| 17 | Enolase, putative, expressed | FP100238 | CX109098 | AY103866 | TA6483_4577999 | TA57592_4565 | TA21609_4558 | TA24081_4547 | TA30958_4513 | TA1054_15368 | TA2047_38727 | TA27204_3702 |
| 18 | glutamine synthetase | FP093960 | TA31026_4530 | TA106794_4577 | TA4495_4577999 | TA54546_4565 | TA20643_4558 | TA25130_4547 | HVCGSA | TA172_15368 | TA1886_38727 | TA29758_3702 |
| 19 | inorganic pyrophosphatase | FP096190 | TA43251_4530 | CO443618 | TA6380_4577999 | TA59108_4565 | TA23897_4558 | TA30783_4547 | TA34581_4513 | TA1185_15368 | DN142995 | TA31096_3702 |
| 20 | ATP carrier protein, mitochondrial precursor | FP099253 | TA35896_4530 | TA102728_4577 | TA5068_4577999 | TA59790_4565 | TA21053_4558 | TA23999_4547 | TA31097_4513 | TA87_15368 | TA2848_38727 | TA33054_3702 |
| 21 | putative 60S ribosomal protein L13E | FP094953 | TA31260_4530 | DV020984 | DV020984 | TA60215_4565 | TA20875_4558 | TA27236_4547 | TA28702_4513 | DV479000 | TA2217_38727 | TA27778_3702 |
| 22 | 20S proteasome beta 5 subunit | FP099328 | TA31509_4530 | TA106951_4577 | TA6673_4577999 | TA66073_4565 | TA22844_4558 | TA27572_4547 | TA28840_4513 | TA491_15368 | DN144611 | TA30587_3702 |
| 23 | hypersensitive-induced reaction protein 3 | FP098624 | TA38205_4530 | TA118573_4577 | AF236375 | TA65393_4565 | TA22857_4558 | TA30317_4547 | TA32753_4513 | TA5_15368 | DN152548 | TA32791_3702 |
| 24 | RAC-ROP-like G-protein | FP097298 | TA46431_4530 | TA114405_4577 | TA12010_4577999 | TA68242_4565 | TA25009_4558 | CA123037 | TA39969_4513 | TA2728_15368 | DN143495 | TA44793_3702 |
| 25 | putative ubiquitin-conjugating enzyme E2 | FP097324 | TA40220_4530 | EB165577 | TA27947_4577999 | CK163054 | TA25004_4558 | TA33158_4547 | TA33470_4513 | TA2347_15368 | TA2437_38727 | TA30654_3702 |
| 26 | cytosolic malate dehydrogenase | FP098036 | TA35551_4530 | TA100021_4577 | TA6641_4577999 | TA56530_4565 | TA21751_4558 | TA25636_4547 | TA31062_4513 | TA119_15368 | TA1938_38727 | TA28353_3702 |
| 27 | putative ribosomal protein S14 | FP093748 | CX108995 | TA107048_4577 | TA5053_4577999 | CK211719 | TA21126_4558 | CA206054 | TA31935_4513 | TA960_15368 | DN150990 | TA32932_3702 |
| 28 | vacuolar ATPase B subunit | FP098688 | TA36775_4530 | TA101378_4577 | TA6474_4577999 | TA66644_4565 | TA21901_4558 | TA26300_4547 | TA31778_4513 | TA999_15368 | TA2057_38727 | TA29709_3702 |
| 29 | ubiquitin-conjugating enzyme | FP099459 | CX105574 | TA94308_4577 | TA531_4577999 | TA50779_4565 | TA21041_4558 | CA078152 | BG369365 | DV471619 | TA2587_38727 | BE037619 |
| 30 | putative eukaryotic translation initiation factor 6 | FP092138 | TA33319_4530 | TA109425_4577 | TA7231_4577999 | TA64994_4565 | TA21108_4558 | TA32315_4547 | TA32638_4513 | TA1178_15368 | DN152014 | TA33895_3702 |
| 31 | S-adenosylmethionine synthetase | FP097379 | TA34161_4530 | TA105895_4577 | TA961_4577999 | TA62083_4565 | TA20698_4558 | TA24506_4547 | TA30795_4513 | TA83_15368 | TA1779_38727 | TA27096_3702 |
| 32 | ribosomal protein L17 | FP098383 | TA35644_4530 | TA103643_4577 | TA4549_4577999 | CA617320 | TA24958_4558 | TA26715_4547 | CV053956 | DV472464 | DN151784 | CB263502 |
| 33 | 60S ribosomal protein L5-1 | FP100793 | TA34574_4530 | TA98568_4577 | TA3434_4577999 | TA62199_4565 | TA21846_4558 | TA25073_4547 | TA31166_4513 | TA914_15368 | TA2137_38727 | TA28926_3702 |
| 34 | ubiquitin fusion protein | FP097720 | CX100967 | CO452718 | TA149_4577999 | CJ550653 | BE599115 | CA247257 | TA29859_4513 | TA1235_15368 | TA1864_38727 | DR215519 |
| 35 | ADP-ribosylation factor | FP093241 | TA34776_4530 | TA95906_4577 | TA1226_4577999 | TA60432_4565 | TA22326_4558 | TA24847_4547 | TA29770_4513 | TA1297_15368 | TA2449_38727 | TA28573_3702 |
| 36 | phosphoglycerate kinase | FP097096 | TA37780_4530 | TA103687_4577 | TA4904_4577999 | TA61760_4565 | TA21050_4558 | TA40512_4547 | TA30174_4513 | TA146_15368 | TA3204_38727 | TA29344_3702 |
| 37 | Elongation factor 1-alpha | FP098428 | TA32605_4530 | TA96825_4577 | TA1919_4577999 | TA54897_4565 | TA21366_4558 | TA24257_4547 | TA29323_4513 | TA34_15368 | TA1738_38727 | TA25834_3702 |
| 38 | putative ribosomal protein S18 | FP101812 | TA36770_4530 | TA106666_4577 | TA2173_4577999 | TA110342_4565 | TA26050_4558 | CA103755 | CV062996 | DV476883 | DN146891 | CB264840 |
| 39 | ATP-citrate synthase, putative, expressed | FP101503 | TA37753_4530 | TA101747_4577 | TA13404_4577999 | TA65690_4565 | TA22224_4558 | TA26656_4547 | TA33463_4513 | TA611_15368 | TA2379_38727 | TA32274_3702 |
| 40 | putative plastidic cysteine synthase 1 | FP095456 | TA37426_4530 | TA97311_4577 | TA11020_4577999 | TA52519_4565 | TA22837_4558 | CA149677 | TA37758_4513 | DV481902 | TA2028_38727 | TA30035_3702 |
| 41 | succinyl-CoA ligase alpha 1 subunit | FP099170 | TA31683_4530 | TA111778_4577 | TA9033_4577999 | TA66033_4565 | TA23393_4558 | CA171198 | TA28925_4513 | TA1033_15368 | TA2616_38727 | TA30769_3702 |
| 42 | reversibly glycosylated polypeptide | FP095202 | TA35355_4530 | TA106903_4577 | TA5866_4577999 | TA63414_4565 | TA22108_4558 | TA25002_4547 | TA28521_4513 | TA73_15368 | TA1818_38727 | TA28985_3702 |
| 43 | small Ras-related GTP-binding protein | FP093553 | TA31718_4530 | CO454767 | TA249_4577999 | CK210723 | TA22310_4558 | TA25295_4547 | TA30645_4513 | TA1018_15368 | DN151472 | TA29161_3702 |
